# Periodization for success—in-season external training loads relative to competition load in American football

**DOI:** 10.3389/fspor.2025.1662240

**Published:** 2025-09-18

**Authors:** Quincy R. Johnson, Yang Yang, Dimitrije Cabarkapa, Dayton Sealey, Shane Stock, Dalton Gleason, Clay Frels, Madi Rink, Andrew C. Fry

**Affiliations:** ^1^ Jayhawk Athletic Performance Laboratory, Wu Tsai Human Performance Alliance, Department of Health, Sport and Exercise Sciences, University of Kansas, Lawrence, KS, United States; ^2^ D2 Lab, Novi Sad, Serbia; ^3^ Physical Activity and Wellness Laboratory, Department of Kinesiology and Sport Sciences, University of Nebraska-Kearney, Kearney, NE, United States; ^4^ Athletics Department, University of Nebraska-Kearney Football, University of Nebraska-Kearney, Kearney, NE, United States; ^5^ Athletics Department, University of Kansas Football, University of Kansas, Lawrence, KS, United States

**Keywords:** sport science, health, performance, GPS, athlete monitoring

## Abstract

**Introduction:**

Despite an exponential development in performance monitoring technologies, the physical performance demands of sport remain an understudied topic in scientific literature. Thus, the primary purpose of this study was to quantify and compare the training loads (TL) of a collegiate American football team between in-season practices and official games by general position group, event type, and to assess the interaction between the two.

**Methods:**

Twenty-seven NCAA Division-II athletes volunteered to participate in this investigation. In-season TL during 32 practices (categorized as days before game day; GD minus) and 11 conference games were recorded using global positioning system technology. Collected data included total duration, total distance, yards traveled per minute, hard running distance, hard running efforts, 2-dimensional (2D) load, and 3-dimensional (3D) load.

**Results:**

A factorial analysis of variance revealed significant main effects in TL for event type (*p* < 0.001) and position groups (*p* < 0.001), and an interaction effect between the two (*p* < 0.001). Unique microcyclic characteristics were observed for each measure of interest. Relative to game values (100%), values for training duration (+25% to −12%; GD-4 to GD-1), yards per minute (+15% to −11%), total distance (+37% to −3%), hard running distance (+33% to −7%), hard running efforts (+33% to −12%), 2D-load (+40% to −7%), and 3D-load (+44% to −3%) were significantly greater than game values on distinct days during the week.

**Discussion:**

These findings can improve the current understanding of practice demands relative to games, which may support more optimal sport-specific periodization approaches within American football.

## 1 Introduction

Despite an exponential development in performance monitoring technologies, the physical performance demands of sport, especially as it relates to effective practice periodization approaches, remains an understudied topic in scientific literature. American football is characterized by a combination of physical, technical, tactical, and psychological characteristics. Optimal health status, body composition, cardiorespiratory fitness, muscular endurance, muscular strength and power, as well as the ability to sprint at maximal speeds and change direction are often characteristics of high performing football athletes (1–5). Foundational research within this sport conducted by Fry and colleagues identified significant differences between the level of play for measures of anthropometrics and physical fitness characteristics such as height, weight, muscular strength and muscular power (2). Furthermore, research conducted by Hoffman et al. examined physical and physiological differences within a cohort of college football starters and redshirt players (5). The authors observed that starters produced significantly higher peak force and peak power during both the squat jump and countermovement jump (CMJ) tasks prior to the start of a game, after each quarter, and following the conclusion of a game. However, changes in peak force and peak power characteristics differed between the groups. Peak force between groups differed only during pre-game testing in favor of starters, while peak power measurements differed at each testing time point in favor of starters. Although this finding highlights physical characteristics related to the role of athletes on a team, more information is needed to develop and translate knowledge related to sport-specific measures of athletic performance and the physiological workloads that these athletes are exposed to, such as those collected by innovative wearable microtechnology devices.

Global positioning system (GPS) units are a popular microtechnology that are often utilized within competitive sport to understand the demands of training and competition as well as to support sport-specific player development, performance, return to play, and return to performance approaches (6–19). GPS systems operate through communication between a wearable sensor and satellites orbiting the earth to provide a measurement of instantaneous movement depending on the sampling frequency. Within the sport of soccer, ample evidence is available which has evaluated training load (TL) metrics during practice sessions as well as official games (11–13, 20–24). External TL metrics typically include a total number of accelerations, decelerations, and distances covered at various velocities and specific time intervals, while internal TL metrics typically include measures of core body temperature, heart rate, caloric expenditure, rating of perceived exertion, session rating of perceived exertion, wellness questionnaires, and recovery scales (6, 18, 24). Although limited, the body of evidence is growing as it relates to GPS implementation and TL monitoring within the American football population (6, 8–10, 14–19, 25).

In a recently published study, Bayliff et al. aimed to determine select physical demands of collegiate American football players during 11 games and to compare such data between playing positions (6). Findings from the study indicated that defensive backs traveled significantly greater distances (approximately 4,500 m) than wide receivers approximately (3,500 m) and offensive linemen (approximately 3,400 m), but not defensive linemen (approximately 400 m). Moreover, significant differences between playing positions were also observed for measures of maximum sprinting velocity, acceleration, and deceleration characteristics showing that the physical demand is higher for defensive back and wide receiver position groups when compared to linemen groups. These findings highlight the position-specific demands of the sport but did not include data from training. In an investigation conducted by Mamon et al, the author's findings revealed that workload intensity metrics were influenced by the interaction between position and drill type within collegiate American football, indicating that distinct position groups exhibited their own unique workload characteristics for the different drill types (10). Additionally, workload intensity (i.e., yards traveled per minute) differed both between drill types and practice days independently. Specifically, greater running intensities were seen in special teams' drills compared with other drill types and Tuesday (i.e., 96 h prior to competition) practice sessions had greater overall intensities compared with other training sessions. These findings undoubtedly contribute to the current body of knowledge. However, a gap in the literature still exists in regard to comparing the physical demands of practice to games as well as the potential identification of a periodization structure implemented within the sport of the American football.

Ensuring that athletes are adequately prepared for competitive demands is a primary goal of the sports performance professional. By utilizing GPS technology and the subsequent data gathered, coaches, sport scientists and sport performance research teams have begun utilizing TL monitoring and periodization models to improve practice structure, volume, intensity, and density to better prepare athletes for competition demands (22–24, 26). One such study of interest includes a recent publication from Stevens et al. which aimed to quantify in-season TL relative to match TL within a cohort of professional soccer players (24). Specifically, the authors reported that relative to match day (MD) values (100%), training values (match day minus four; MD-4, to match day minus one; MD-1) for running (−52% to −20%; MD-4 -MD-1) and high-speed running (−38% to −15%) were lower when compared to total distance (−67% to −35%), and all considerably lower than MD values. Conceptually, this approach should and can be applied to the American football population to understand and optimize training approaches designed for preparing the athlete for optimal performance by leveraging wearable microtechnology such as GPS units.

Therefore, the primary aim of the present study was to quantify and compare the external TL of a successful collegiate American football team's practices and games by position group, day of practice, and to assess the interaction between the two. Based on prior findings, the authors hypothesized that significant main effects would be observed between position groups and practice days for the variables of interest, and that a significant interaction would be observed between the two due to unique positional demands (6, 8–10, 14–19, 25). A secondary aim of the present study was to conduct a subgroup analysis to compare GPS-derived variables between starters and non-starters and between athletes who continued their playing career at the professional level and those who did not, both at the group level and across game days. The final aim of the present study was to contribute evidence-based information related to an understudied and underrepresented level of competition to bridge a gap in knowledge.

## 2 Materials and methods

### 2.1 Participants

27 NCAA Division-II American football athletes (mean ± SD; height: 185.2 ± 5.3 cm; body mass: 94.2 ± 16.3 kg; age = 22.3 ± 1.1) participated in the study. According to McKay et al., this cohort of athletes would be classified as “Tier 3: Highly Trained/National Level” which only includes approximately 0.014% of the global population (27). All subjects participated in regular training sessions administered by their respective sports performance coaches and were free of musculoskeletal injuries. Further inclusion criteria for this study only included individual observations if athletes participated in at least 60% of the 11 conference games, which are similar to prior reported methods at the NCAA Division-I level (6). The average number of games played for athletes included within this study were 10 out of 11 (or 90.1%) for the cohort. Since data related to snap counts and on-field minutes were not available for this sport or level of competition, the authors utilized the aforementioned method to reduce the sample size from 34 to include only players with high game involvement. To achieve the secondary aim of this study, athletes were organized into subgroups of starters (*n* = 14) and non-starters (*n* = 13), as well as athletes who continued their career at the professional level (*n* = 5) vs. those who did not (*n* = 22). This study was conducted in accordance with the Declaration of Helsinki and was approved by the university's Institutional Review Board (031022-1).

### 2.2 Procedures

TL data were collected for all athletes using a GPS system (specifications below) during the in-season competitive period. All practices included a warm-up, individual and group technical development focus, as well as group and team tactical strategy focus. The team played one game per week. A typical week included three days of practice, one walkthrough (i.e., training session with limited physical contact), and one game with six full days between the games. Regular practice sessions (32 in total) were subsequently categorized as game day minus four (GD-4; 96 h prior to competition), game day minus three (GD-3; 72 h prior to competition), and game day minus two (GD-2; 48 h prior to competition). Game day minus one (GD-1; 24 h prior to competition) was programmed as a walkthrough day and GPS units were not worn.

### 2.3 Data collection and analysis

The TL testing methodology was adapted from previous research reports (6, 10, 19, 25, 30–33). Athletes wore the GPS unit (Sports Performance Tracking, 10 Hz GPS, 10 Hz 3D accelerometer, 10 Hz 3D gyroscope; 10 Hz 3D magnetometer, Melbourne, Australia), during every official practice (*n* = 32) and competition (*n* = 11) throughout the respective football season spanning over 11 weeks (regular season only). Each unit was fully charged before being positioned in a customized pouch that was located between participant's scapulae as part of a vest made of nylon and elastane to be worn under the shoulder pads. These pouches were specifically designed by the company to prevent unnecessary movement of the sensors, and the location of each sensor remained consistent throughout the course of the season. In no way did the monitor or vest restrict the athlete's movement during athletic play. Data were downloaded and processed after each event. A previous research report has suggested sufficient validity of this system in tasks, such as walking, jogging, sprinting, as well as jumping and changing directions (28). Similar to Mamon et al., session recordings during practices and games were started and ended at the same time for each athlete on the team (10). Although sessions were further broken down by phases (e.g., halves and quarters), for the interests of this study, the cumulative external TL of each session recording was used for research purposes.

### 2.4 Data acquisition

The variables of interest for this study were training duration (minutes), total distance (yards), hard running distance (yards), hard running efforts (number or instances), yards per minute (otherwise known as work rate), as well as 2D and 3D (arbitrary units) loads. The load variables were calculated through a widely accepted formula, and it captured all movements in the x, y, and z axes, quantifying load sustained from motion, jumps, and impacts. Throughout literature examining sports performance metrics derived from GPS systems, this metric is often referred to as “total player load” and can be critical for better understanding the physical workload of American football athletes (6, 9, 10, 20). Operational definitions for each variable of interest were provided by the technology company, are similar in nature to previous literature, and can be found in Table 1.

**Table 1:** Operational definitions for variables of interest.

Variable	Operational definition
Duration (minutes)	Total duration of event.
Distance (yards)	Total distance covered during event.
Yards per minute (yards)	Total distance traveled relative to the event time.
Hard running distance (yards)	Total distance traveled at a velocity of >4.5 m/s (10.1 mi/h).
Hard running efforts (number)	Total running instances completed within the hard running threshold.
2-dimensonal load (au)	Total forces in the x- and y- axes during event.
3-dimensional load (au)	Total forces in the x-, y-, and z-axes during event.

### 2.5 Statistical analysis

Descriptive statistics, means, and standard deviations were calculated for each variable. A between 4 [event] × 3 [position group] factorial analysis of variance was conducted to compare the main effects of event and position group and the interaction effect between event and position group on the dependent variables of interest. The event type included four levels (GD-4, GD-3, GD-2, GD) and position group consisted of three levels (Linemen, Big Skill, Skill). Independent samples *t*-tests were conducted for two additional subgroup analyses which (1) compared GPS-derived measures for starters vs. non-starters and (2) compared athletes who continued their careers professionally vs. those whose career concluded at the collegiate level. Semantics and classification of subjects can play a critical role in the communication of research findings. GD (game day) rather than the commonly used MD (match day) was selected for an abbreviation because American football athletes compete in games rather than matches. The classification of specific position groups into general position groups was designed to align with the common practice of strength and conditioning programs who organize their athletes into these groups as well as to improve the translation of the findings of this investigation to the sports performance professional. Alpha (*α*) levels for all statistical testing were set at *p* ≤ 0.05 as the acceptable level of significance. Bonferroni *post hoc* tests were performed to determine the specific location of significance among the independent variables. *A priori* power analysis using G*Power software version 3.1.9.7 was conducted to determine the necessary statistical power. Statistical analyses were performed using IBM Statistical Package for the Social Sciences (IBM SPSS for Windows, version 24; SPSS, Inc., Chicago, IL, USA).

## 3 Results

Descriptive data (mean ± SD) of practices and games are presented in Tables 2, 3. Significant main effects analysis showed that there is a significant difference between the three types of general position groups, Linemen, Big Skill, and Skill for measures of total distance [F(2, 1,562) = 22.9, *p* < 0.001], yards per minute [F(2, 1,562) = 90.7, *p* < 0.001], hard running distance [F(2, 1,562) = 226.5, *p* < 0.001], hard running efforts [F(2, 1,562) = 241.2, *p* < 0.001], 2D load F[2, 1,562] = 26.3, *p* < 0.001), and 3-dimensional load F[2, 1,562] = 32.6, *p* < 0.001). No significant differences between general position groups were observed for measures of total duration [F(2, 1,562) = 0.1, *p* = 0.91]. Significant main effects analysis revealed a difference between the four types of events, GD-4, GD-3, GD-2, and GD for measures of total duration [F(3, 1,562) = 51.3, *p* < 0.001], total distance [F(3, 1,562) = 61.8, *p* < 0.001], yards per minute [F(3, 1,562) = 36.1, *p* < 0.001], hard running distance [F(3, 1,562) = 5.7, *p* < 0.001], hard running efforts [F(3, 1,562) = 7.5, *p* < 0.001], 2D load [F(3, 1,562) = 49.5, *p* < 0.001], and 3D load [F(3, 1,562) = 57.5, *p* < 0.001].

**Table 2:** External training load metrics (mean ± SD) and comparison statistics between events.

Variable	GD-4	GD-3	GD-2	Game
Absolute values				
Duration (min)	147.2 ± 8.8*^,^**	143.0 ± 9.8*^,^**	102.9 ± 30.8*	117.4 ± 51.2
Distance (yards)	5,849.2 ± 691.5*^,^**	5,890.3 ± 810.4*^,^**	4,173.4 ± 799.4	4,301.2 ± 2,088.8
Yards per minute (yards)	39.8 ± 4.4*	41.2 ± 5.1*	41.4 ± 7.2*	35.8 ± 7.2
Hard running distance (yards)	282.9 ± 185.1	313.7 ± 193.8*^,^**	219.9 ± 168.6	236.5 ± 178.8
Hard running efforts (number)	14.2 ± 9.2**	15.9 ± 9.8*^,^**	10.6 ± 7.2	12.0 ± 8.8
2-dimensonal load (au)	217.7 ± 53.1*^,^**	222.0 ± 55.7*^,^**	147.0 ± 45.4*	158.7 ± 83.9
3-dimensional load (au)	344.9 ± 73.8*^,^**	353.1 ± 79.8*^,^**	238.2 ± 70.1	245.5 ± 130.3
Relative values				
Duration (min)	1.3 ± 0.17*^,^**	1.2 ± 0.2*^,^**	0.88 ± 0.6*	1.00
Distance (yards)	1.4 ± 0.33*^,^**	1.4 ± 0.4*^,^**	1.0 ± 0.4	1.00
Yards per minute (yards)	1.1 ± 0.61*	1.2 ± 0.7*	1.2 ± 1.0*	1.00
Hard running distance (yards)	1.2 ± 1.04	1.3 ± 1.1*^,^**	0.9 ± 0.9	1.00
Hard running efforts (number)	1.2 ± 1.05**	1.3 ± 1.1*^,^**	0.9 ± 0.8	1.00
2-dimensonal load (au)	1.4 ± 0.63*^,^**	1.4 ± 0.7*^,^**	0.9 ± 0.5*	1.00
3-dimensional load (au)	1.4 ± 0.57*^,^**	1.4 ± 0.61*^,^**	1.0 ± 0.5	1.00

AU, arbitrary unit; Relative values = 1.00 = 100% of game values.

* Significantly different when compared to game (*p* < 0.05).

** Significantly different when compared to GD-2 (*p* < 0.05).

**Table 3:** External training load metrics (mean ± SD) and comparison statistics between general position groups.

Variable	GD-4	GD-3	GD-2	Game
Linemen (*n* = 3)				
Duration (min)	144.9 ± 11.6*^,^**	141.41 ± 9.9*^,^**	100.6 ± 12.2	119.0 ± 49.7
Distance (yards)	4,790.0 ± 532.9**	4,718.2 ± 551.9**	3,355.2 ± 582.7	3,987.0 ± 1,838.4
Yards per minute	33.1 ± 2.5	33.4 ± 3.4	33.3 ± 3.8	33.0 ± 6.4
Hard running distance (yards)	20.8 ± 24.7	20.2 ± 18.6	16.4 ± 19.9	61.9 ± 49.5
Hard running efforts	0.8 ± 1.4	0.8 ± 1.3	0.6 ± 1.1	3.0 ± 2.7
2-dimensonal load (au)	166.3 ± 42.7**	169.2 ± 20.7**	104.5 ± 32.6*^,^**	155.8 ± 77.1
3-dimensional load (au)	256.9 ± 64.2**	263.1 ± 28.5**	165.6 ± 51.2*^,^**	229.4 ± 112.8
Big skill (*n* = 9)				
Duration (min)	147.8 ± 8.0*^,^**	143.3 ± 9.6*^,^**	103.3 ± 35.9	115.1 ± 53.7
Distance (yards)	5,937.9 ± 530.0*^,^**	6,025.4 ± 627.6*^,^**	4,465.1 ± 696.3	4,431.3 ± 2,217.0
Yards per minute	40.2 ± 3.3*^,^**	42.1 ± 3.8*	44.5 ± 6.3*	37.4 ± 7.9
Hard running distance (yards)	215.6 ± 88.3*	261.6 ± 88.7	216.4 ± 112.0*	273.8 ± 186.9
Hard running efforts	11.3 ± 5.2	13.5 ± 5.1	11.1 ± 5.8	13.3 ± 8.8
2-dimensonal load (au)	212.2 ± 36.7*^,^**	220.6 ± 44.4*^,^**	153.3 ± 38.9	156.4 ± 88.0
3-dimensional load (au)	347.0 ± 40.4*^,^**	360.1 ± 56.3*^,^**	256.5 ± 56.6	248.1 ± 139.1
Skill (*n* = 14)				
Duration (min)	147.3 ± 8.4*^,^**	143.1 ± 9.8*^,^**	103.3 ± 35.9*	118.6 ± 50.0
Distance (yards)	6,054.4 ± 579.0*^,^**	6,078.8 ± 741.0*^,^**	4,167.1 ± 778.4	4,283.2 ± 2,049.4
Yards per minute	41.2 ± 3.9*	42.5 ± 4.5*	41.2 ± 6.9*	35.3 ± 6.7
Hard running distance (yards)	393.7 ± 167.3*^,^**	420.4 ± 181.8*^,^**	269.5 ± 183.5	248.9 ± 170.0
Hard running efforts	19.5 ± 8.0*^,^**	21.1 ± 8.9*^,^**	12.6 ± 6.9	13.0 ± 8.6
2-dimensonal load (au)	234.3 ± 55.9*^,^**	235.7 ± 60.8*^,^**	152.5 ± 47.0	160.7 ± 82.8
3-dimensional load (au)	365.4 ± 77.7*^,^**	370.0 ± 87.8*^,^**	242.8 ± 71.8	247.1 ± 127.8

AU, arbitrary unit.

* Significantly different when compared to game (*p* < 0.05).

** Significantly different when compared to GD-2 (*p* < 0.05).

Significant interaction effects between position and event were observed for measures of total distance [F(6, 1,562) = 2.3, *p* = 0.034], yards per minute [F(6, 1,562) = 8.5, *p* < 0.001], hard running distance [F(6, 1,562) = 20.1, *p* < 0.001], hard running efforts [F(6, 1,562) = 17.0, *p* < 0.001], 2D load [F(6, 1,562) = 4.6, *p* < 0.001], and 3D load [F(6, 1,562) = 4.2, *p* < 0.001]. No significant interaction effects between position and event day were observed for measures of duration [F(6, 1,562) = 0.2, *p* = 0.98].

**Figure 1:**
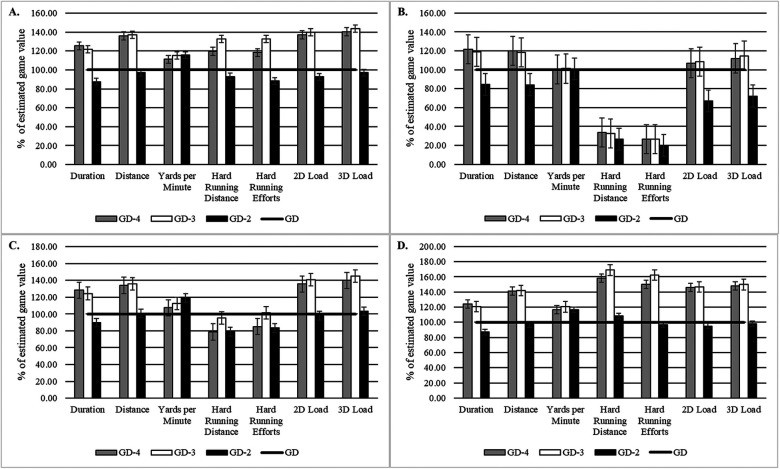
Microcylic periodization characteristics of a typical week with three practices (GD-4, GD-3, and GD-2) and one game (GD). A typical week with 1 game was considered to be a week with a recovery training day (day after the game; not recorded), a day off, four subsequent training sessions [GD-4, GD-3, GD-2 and GD-1 (not recorded)] and a game. GD-4 = practice session 4 days before game day (GD) for the **(A)** sample, **(B)** Linemen, **(C)** Big Skill, **(D)** Skill.

Illustrative data for practices and games are presented in Figure 1. For the sample, relative to game values (100%), practice values for total durations were between +25% and −12% (range GD-4–GD-2), total distances were between +37% and −3%, yards per minute were between +15% to +11%, hard running distances were between +33% and −7%, hard running efforts were between +33% and −12%, 2-dimensional loads were between +40% and −7%, and 3-dimensional loads were between +44% and −3%. Abbreviations: GD-4 = practice session 4 days before game day (GD).

For Linemen, relative to match values (100%), practice values for total durations were between +22% and −15% (range GD-4–GD-2), total distances were between +20% and −16%, yards per minute were between +1% and 0%, hard running distances were between −74% and −67%, hard running efforts were between −80% and −73%, 2-dimensional loads were between +9% and −33%, and 3-dimensional loads were between +15% and −28%. Abbreviations: GD-4 = practice session 4 days before game day (GD).

For Big Skill, relative to match values (100%), practice values for total durations were between +28% and −10% (range GD-4–GD-2), total distances were between +36% and +1%, yards per minute were between +19% and +7%, hard running distances were between −4% and −21%, hard running efforts were between −4% and −17%, 2-dimensional loads were between +41% and −2%, and 3-dimensional loads were between +45% and +3%. Abbreviations: GD-4 = practice session 4 days before game day (GD).

**Figure 2:**
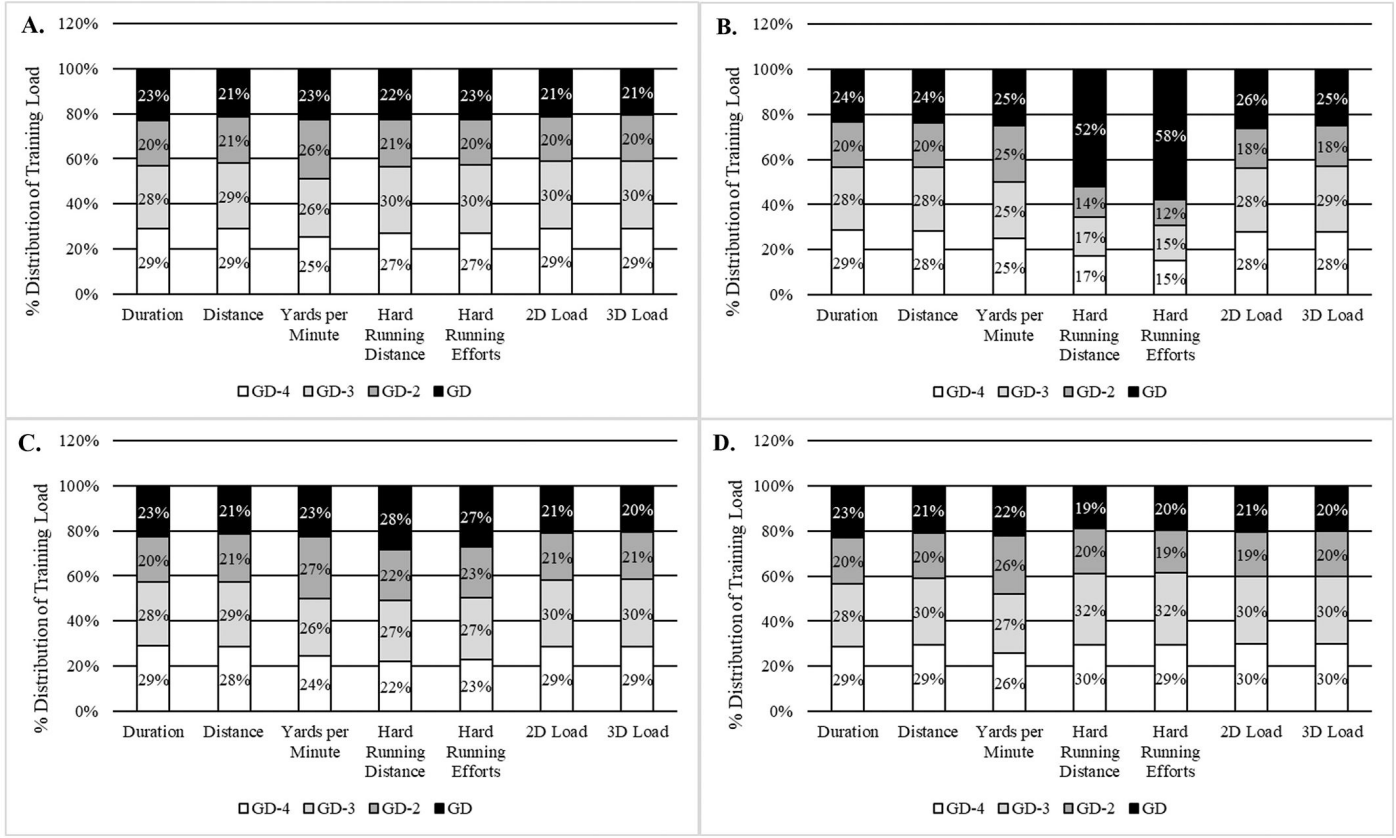
Cumulative weekly training load of a typical week with one game and three subsequent practices (GD-4, GD-3, and GD-2). A typical week with 1 game was considered to be a week with a recovery training (day after the game; not recorded), a day off, four subsequent training sessions [GD-4, GD-3, GD-2 and GD-1 (not recorded)] and a game. GD-4 = practice session 4 days before game day (GD) for the **(A)** sample, **(B)** Linemen, **(C)** Big Skill, **(D)** Skill.

For Skill, relative to match values (100%), practice values for total durations were between +24% and −13% (range GD-4–GD-2), total distances were between +42% and −3%, yards per minute were between +20% and +17%, hard running distances were between +69% and +8%, hard running efforts were between +62% and −3%, 2-dimensional loads were between +47% and −5%, and 3-dimensional loads were between +50% and −2%. Abbreviations: GD-4 = practice session 4 days before game day (GD). Training load compositions and distributions are presented in Figure 2.

**Figure 3:**
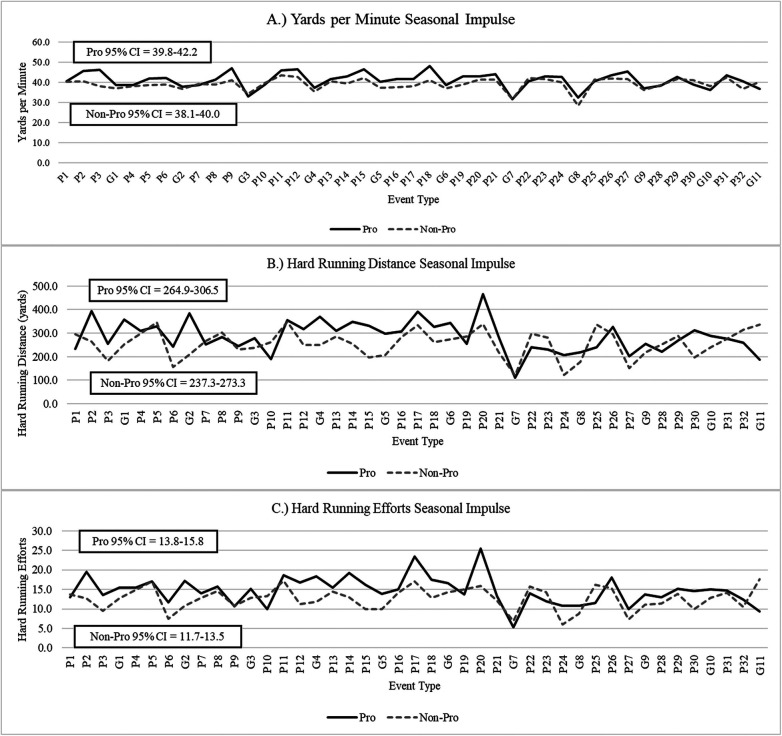
Exploratory seasonal impulse for changes in measures of **(A)**, yards per minute **(B)**, hard running distance, and **(C)** hard running efforts between starter and non-starter subgroup.

**Figure 4:**
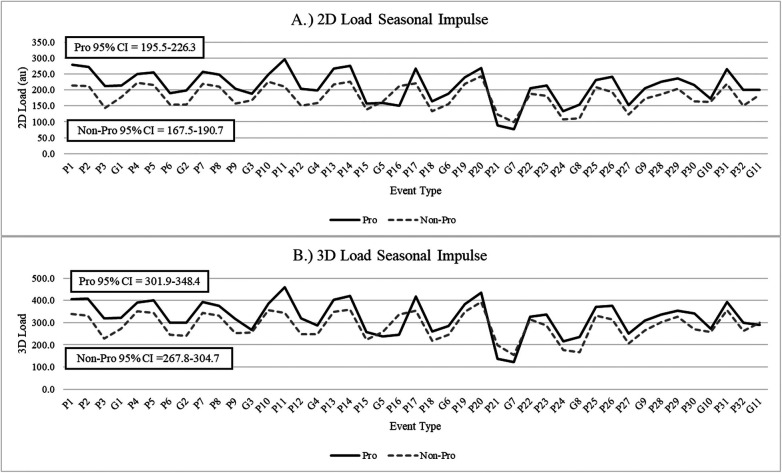
Exploratory seasonal impulse for changes in measures of **(A)** 2D load and **(B)** 3D load between professional and non-professional subgroups.

When analyzed by subgroup, starters had significantly higher measures of yards per minute (38.7 ± 6.9, 37.2 ± 7.2) when compared to non-starters (*p* < 0.01). However, non-starters had significantly higher measures of hard running distance (283.9 ± 191.0, 235.7 ± 178.1) and hard running efforts (13.8 ± 8.9, 12.1 ± 9.1) when compared to starters (*p* < 0.01). No significant differences were observed for measures of total duration, total distance, 2D load, or 3D load between starters and non-starters (*p* > 0.05). When analyzed by professional and non-professional subgroups, athletes who continued their careers professionally had significantly higher measures of yards per minute (39.3 ± 6.4, 37.8 ± 7.2), hard running distance (286.9 ± 159.7, 245.9 ± 189.8), hard running efforts (14.4 ± 8.1, 12.3 ± 9.2), 2D load (198.9 ± 83.3, 170.8 ± 74.4), 3D load (304.2 ± 124.8, 270.8 ± 116.5) when compared to those who did not (*p* < 0.01). It should be noted that two out of the five athletes within the cohort of athletes who continued their careers professionally were not classified as starters. Could there be an underlying athletic performance characteristic that contributed to this? To further explore these findings and this question, Figures 2, 3 and 4 were created to illustrate season long changes in measures that significantly differed between professional and non-professional subgroups. These findings have been termed as seasonal impulse, which provides a measure of the outputs of interest (e.g., hard running distance, hard running efforts, etc.) relative to time (e.g., days).

## 4 Discussion

The primary purpose of the present study was to quantify and compare external TL between practices and games measured via GPS technology for NCAA Division-II American football athletes. In comparison to the NCAA Division-I level, the NCAA Division-II level of competition is often underrepresented in the scientific literature, and this study contributes to bridging a knowledge gap. Significant differences in the selected measures of interest were observed for position group, event type, and the interaction between the two. Based on the results, it is within reason to conclude that the original hypothesis was supported. Specifically, a significant main effect was observed when comparing total performance duration, total distance, total hard running distance, total hard running effort duration, work rate, 2D load, and 3D load between the position groups (line, big skill, and skill) and between days measured (GD-4, GD-3, GD-2, and GD). Significant interaction effects between position and day were observed as well, which further demonstrates the unique physical demands of each position group. By translating perspectives, approaches, and findings from soccer, the authors have further contributed to the field's current understanding of the physical demands of American football and addressed a critical topic of interest, load monitoring via GPS technology. The identification and quantification of microcyclic periodization characteristics may be valuable to the sports performance professional who can utilize this data to guide sport-specific approaches that better prepare athletes for the physical demands of training, competition, and optimal athletic performance, a key feature of sports science research.

Measures of duration, total distance, yards per minute, hard running distance and efforts, as well as 2- and 3-dimensional load are commonly reported in sports performance literature and help sports performance practitioners further understand the physical demands of practice and competition (6, 10–26, 29). Significant differences between the three types of general position groups, Linemen, Big Skill, and Skill for measures of total distance on GD-4, GD-3, GD-2 were observed. Linemen covered significantly less total distance when compared to Big Skill and Skill position groups, yet no significant differences between Big Skill and Skill position groups were detectable across days. However, no significant differences were observed between positions groups on GD for measures of total distance. Beyond the expected differences, the non-significant findings highlight the homogeneity of competition demands at the NCAA Division-II level of competition regardless of position as well as the need for sports performance practitioners to ensure that athletes can repeatedly meet these demands throughout the course of the season. Furthermore, these findings generally align with those reported by Bayliff et al. who examined the physical demands of NCAA Division-I football players during games and Mamon et al. who examined similar metrics during training at the same level of competition (6, 10). However, it is important to note that when compared to NCAA Division-I football athletes, the GPS-derived measures at the NCAA Division-II level are lower. These observations may be due to differences in performance characteristics, physical demands, or scheduling structure between divisions but should be further investigated and published in the future. Although directly comparing competition levels was not a primary purpose of this study, it contributes to current perspectives in the field and data across levels of competition should be interpreted cautiously.

Findings from this study suggested that external training loads increased earlier in the week before decreasing in practices closer to games (GD-2), and the relative cumulative external training load for the days before game day was higher than those from games. When comparing position groups, measures of total distance, hard running distance, hard running effort, yards per minute, 2D load, and 3D load were significantly different, and confirmed our hypotheses. However, yards traveled per minute were similar to or greater than competition demands, which may be (1) a key contributor to adequately ensuring athletes are prepared for competition and (2) a key feature of this individual team's approach towards practicing (e.g., short duration-high intensity vs. long duration-high volume). Hard running distance between all positions significantly differed on all days except for GD where no significant difference was detected between Big Skill and Skill. Hard running efforts between all positions significantly differed on all days except for GD where no significant difference was detected between Big Skill and Skill. 2D load between Linemen and Big Skill and Linemen and Skill on GD-4, GD-3, and GD-2 were significantly different. However, no significant differences were observed between position groups on GD which highlights the demands of competition as well as the importance of properly preparing athletes to withstand those demands. Significant differences were observed for measures of 3D load between Linemen and Big Skill and Linemen and Skill on GD-4, GD-3, and GD-2. On GD however, no significant differences were observed between Big Skill and Skill. Although periodization will be discussed further in the following paragraph, the strategy utilized to prepare athletes within this cohort often included exposures to training volumes (i.e., total duration, total distances, 2D loads, and 3D loads), intensities (i.e., hard running densities and hard running efforts), and densities (i.e., yards per minute) above that of competition demands. Although this form of analyses is common within soccer, to the author's knowledge this is among the first published analyses to this extent within American football. To add, other reports in soccer have shown vast differences in external load periodization strategies in training prior to games, comparatively (22–24, 26). Whereas external loads within soccer during a training week may be lower than competitive demands, this approach is likely influenced by the unique scheduling characteristics of the sport. Furthermore, these findings may be due to positional demands, training philosophy, travel, etc. but highlights the need for this type of analysis across competitive sports, specifically American football.

Understanding the periodization of training load and how it compares to competition demands can enhance practitioners' understanding of sport-specific cumulative training load composition and distribution. Hard running distance and frequency are two commonly monitored external TL metrics within American football (6, 10). Cumulative measures of training load were found to display a uniform periodization structure at the sample level where GD-4 and GD-3 were similar to one another (28% distribution on average), but significantly higher for most metrics when compared to GD-2 and GD (22% distribution on average). However, at the general position group level the microcyclic periodization characteristics for the Linemen group were unique for hard running distance and hard running efforts where this position only accumulated 48% and 42% of this specific volume throughout GD-4 to GD-2, compared to GD where 52% and 58% of the load was accumulated. For these exact measures, the Big Skill group accumulated 72% and 73% throughout GD-4 to GD-2, compared to GD where 28% and 27% were accumulated. Additionally, the Skill group accumulated 81% and 80% throughout GD-4 to GD-2, compared to GD where 19% and 20% were accumulated for measures of hard running distance and hard running efforts. The authors posit that the types of drills implemented, foci of practice, positional demands, and competition demands may have contributed to this unique observation but should be monitored and adapted in the future to ensure that athletes are adequately prepared for the demands of competition. Furthermore, it appears that the periodization of practice training loads at the microcyclic level, although unique to position groups, may be most suitable for American football. Mamon et al. investigated position-specific external training load among collegiate American football athletes and found significant position differences in intensity-related metrics which aligns with our findings and may provide further explanation based on drill intensity (10). Similar results have been found in previous studies that have quantified training load in other team sports. For instance, in soccer match day minus 4 (MD-4) the highest loads were observed during the six-day training period in comparison to match day (22–24). Furthermore, this periodization approach has also been reported for starters vs. non-starters and across age groups (in relation to an increase in athletic ability) (23). These findings somewhat align with ours, with the exception being measures of hard running distance and hard running efforts in the Linemen position group. Often overlooked, Linemen play a critical role in the success of a team. Additionally, as the sport of American football advances towards a more dynamic style of play both technically and tactically, further attention should be devoted to understanding this populations physiology as well as physical performance characteristics in an effort to enhance health and peak performance. To conclude, a lack of research within American football populations warranted this study to be conducted, as training load composition and distribution may be influenced by sport, competitive level, position group, coaching philosophy, and schedule characteristics.

Within soccer, external training load variables have been regularly used as an athlete monitoring tool for sports practitioners. Framework approaches have suggested utilizing external loads as a means of both assessment of performance and injury risk mitigation via periodization protocols (i.e., undulating and linear periodization) (29). Ravé et al. proposed using predetermined parameters calculated via workload equations in reference to game day values for daily, weekly and monthly planning, and their programming offers a guide for analyzing and prescribing external loads at both the team and individual level (12). Although further research is warranted for the translatability of such equations, it is possible that they could be used as a guideline for establishing predicted training load in an attempt to periodize practices within American football to optimize preparedness for competition.

As with any research, this study has its limitations. For transparency purposes, the authors have shared those here to contribute to future, more discrete research projects within the field and related to the sport of American football. First, the sample size of the present study was smaller (*n* = 27) than preferred which may not represent the entire population well. For example, an ideal sample size of a team that has a population size of 85 would be 70 athletes if a confidence level of 95% is expected. This would allow for stronger comparisons between subgroups. However, there are several limitations related to affordability of technology, integration of technology, as well as data management and translation over a longer period for a sample of that magnitude which may make this research methodology more difficult to attain. Next, external training load quantification between levels of competition may differ based on the physiological and physical demands of sport, type of technology used and its capacity to allow for more discrete filtering methods and overall access to resources and personnel to carefully collect data. Thus, our quantification utilizing NCAA Division-II American football athletes may not share similar traits in other levels of competition, such as NCAA Division-I and the National Football League (NFL) due in part to these reasons. Future research should expand upon this study by standardizing data collection process, monitoring training loads via objective and subjective variables within the same study, and completing descriptive studies on external training loads at different competitive levels (e.g., a collaborative study that includes amateur, collegiate, and professional athletes). Additionally, observing external training load characteristics on GD-1 would contribute to a more complete understanding of the cumulative load experienced by the American football athlete throughout a typical week in-season. Within this study, the hard running thresholds utilized were recommended by the hardware company specifically for the sport of American football but diverge from what has been reported previously which may be a limitation for comparison purposes. Future research should aim to standardize thresholds based on the sport, athlete's ability, competition level, or a combination of each. Finally, observing potential differences between non-starters and starters may give practitioners better insight into how training loads may need to be programmed for different individuals depending on active playing time and developmental goals. This could be further be categorized into specific position groups to accommodate specific differences based on positional demands (e.g., higher accelerations or decelerations, higher total distances, etc.). As the utilization of GPS-derived external loads gains popularity among sports science and strength and conditioning professionals, it may be beneficial to begin establishing optimal periodization strategies to prepare for game day. Quantifying these loads is ultimately the first step.

## 5 Conclusion

In conclusion, the findings of this study quantified and compared external training loads during practice and games to further the translation and applicability of commonly collected data. Observations from this study suggest that significant differences in external TL variables that measure volumes, intensities, and densities between practice and games exist and can be utilized to enhance athlete preparation and performance. For the strength and conditioning practitioner, work rate or training density should be of particular interest as time allowed for training is often a common constraint. Relative to time, strength and conditioning professionals may be able to design training regimes that increase the translation of physical fitness to athletic performance by prescribing similar work rate/training densities to mimic the specific metabolic demands of training and competition. Further attention should also be given to the periodization of external TL variables at each cyclical level (i.e., macro-, meso-, and microcycle). These findings can be utilized by sport performance professionals to plan and implement optimal practice volumes, intensities, and densities in a periodized fashion prior to games.

## Data Availability

The raw data supporting the conclusions of this article will be made available by the authors, without undue reservation.
